# A Case of Radiation-Induced Aortitis in a Patient With Cervical Cancer

**DOI:** 10.7759/cureus.35484

**Published:** 2023-02-26

**Authors:** Cédric Charrois-Durand, Marie-Claude Beauchemin, Maroie Barkati

**Affiliations:** 1 Department of Radiation Oncology, Centre Hospitalier de l'Université de Montréal (CHUM), Montréal, CAN

**Keywords:** radiation induced vasculitis, recurring cervical cancer, complication of treatment, side effects of radiotherapy, abdominal aortitis, chatgpt

## Abstract

Radiation-induced aortitis is a rare but potentially serious complication of radiotherapy. We report the case of a 46-year-old female with a history of cervical cancer who developed radiation-induced aortitis following two courses of concurrent chemoradiation. The patient was asymptomatic, and the condition was detected during a routine follow-up positron emission tomography (PET) scan. The patient was referred to rheumatology for differential diagnosis, which ruled out non-radiation-induced aortitis. The condition was managed conservatively, and a follow-up computed tomography (CT) scan showed resolution of the aortitis but the progression of aorto-iliac fibrosis. The patient was then started on prednisone, which led to a regression of the aorto-iliac vessel thickening.

## Introduction

Radiation-induced aortitis is a rare but potentially serious complication of radiotherapy. It usually appears more than 10 years after radiotherapy [1], sometimes sooner [2]. Its exact incidence is unknown. Risk factors for radiation-induced arterial complications include dose, field size, hyperlipidemia, diabetes, hypertension and smoking [2]. This form of vasculitis occurs when radiation damages the vascular endothelium, leading to inflammation of the aortic wall (Abstract: Girinsky T. Effects of Ionizing Radiation on the Blood Vessel Wall, 2000). The condition can be asymptomatic or present with a range of symptoms, including fever, malaise, abdominal or back pain, and ischemic symptoms [1,3]. We present a case of radiation-induced aortitis that occurred nine years after radiation therapy.

## Case presentation

**Figure 1:**
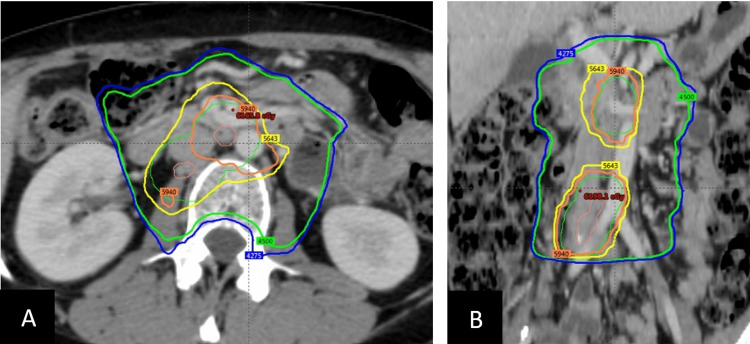
Axial (A) and coronal (B) planning CT scan illustrating the dose distribution of her second radiation course. The planning target volume is depicted in green, inside the yellow isodose line.

A 46-year-old female smoker with a history of cervical cancer presented for a routine follow-up in September 2020. The patient had undergone two courses of concurrent chemoradiation. First, in 2005 for a Fédération internationale de gynécologie et d'obstétrique (FIGO) stage IB1 squamous cell carcinoma that showed positive lymphovascular invasion and positive obturator nodes post radical hysterectomy and bilateral pelvic node dissection, when she only received 41.4 Gy of the 45 Gy prescribed since she developed treatment induced intractable diarrhea. Second, in 2012 when she developed a nodal recurrence in the paraaortic region and received 45 Gy in 25 fractions to the lombo-aortic field with a sequential boost of 14.4 Gy in 8 fractions on the positive nodes (total dose of 59.4 Gy in 33 fractions). The treatment field for this course is depicted in Figures 1A, 1B). Both treatment courses were delivered using intensity-modulated radiation therapy (IMRT).

**Figure 2:**
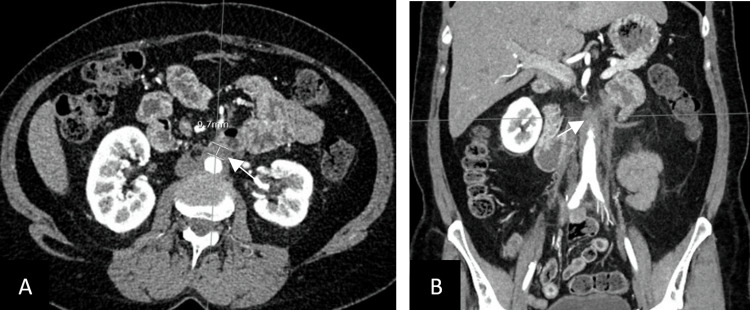
Axial (A) and coronal (B) slices of CT angiogram depicting thickening and infiltration of the abdominal aorta (white arrows) with periaortic fat infiltration.

In 2018, she developed a radiation-induced enteritis that was treated with surgery. She also received two chemotherapy courses in 2018 and 2019 for disease recurrence and progression. During follow-up, a positron emission tomography (PET) scan done in September 2020 revealed moderately intense uptake of the infrarenal abdominal aorta over 5 cm that was compatible with active vasculitis. An angio-computed tomography (CT) scan done shortly after showed thickening and infiltration of the abdominal aortic wall from 1 cm below the renal artery to the iliac bifurcation, associated with periaortic fat infiltration, without dissection or thrombus (Figures 2A, 2B). She was asymptomatic.

The patient was referred to rheumatology for differential diagnosis, which ruled out non-radiation-induced aortitis. Radiation oncology concluded that the phenomenon could be radiation-induced since it occurred “in field.” No corticosteroid therapy was initiated because of the absence of clinical consequences of the radiological findings.

**Figure 3:**
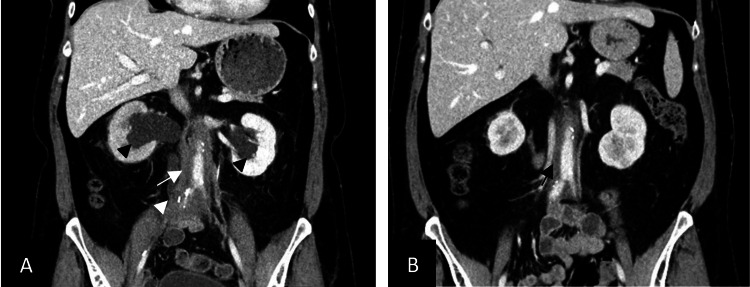
Coronal CT scan slices depicting evolution of aortitis to fibrosis (A) (white arrow) and regression of fibrosis after corticosteroid therapy (B) (black arrow). Also note the right ureteritis (white arrowhead) and bilateral hydronephrosis (black arrowheads).

A follow-up CT scan in early 2021 showed resolution of the aortitis with progression of aorto-iliac fibrosis without other signs of acute vasculitis. The patient was also diagnosed with right periureteral infiltration compatible with ureteritis (Figure 3A). Hydronephrosis was present and treated with percutaneous nephrostomy. Vascular surgery and interventional radiology were consulted, but biopsy of the periaortic fibrosis was deemed too risky. Internal medicine initiated empirical prednisone in April 2021, and a follow-up CT scan in June 2021 showed regression of the circumferential thickening of the aorto-iliac vessels (Figure 3B).

## Discussion

Radiation-induced aortitis is a rare but potentially serious complication of radiotherapy. The condition typically occurs several years after radiation therapy and can present with a range of symptoms [1]. In some cases, radiation-induced aortitis may present as an acute complication, with symptoms such as fever, chest or abdominal pain, and signs of systemic inflammation [1,3]. However, in many cases, radiation-induced aortitis may be asymptomatic, and the diagnosis may be made incidentally on imaging studies obtained for other reasons. It is worth noting that the risk of developing radiation-induced aortitis depends on several factors, including the total radiation dose, the fractionation schedule, the field size, the age of the patient at the time of radiation therapy, and underlying risk factors such as diabetes, smoking, hypertension, and hyperlipidemia [2]. However, close follow-up with imaging studies is not recommended for all patients with a history of radiation therapy to the aorta because the incidence of symptomatic radiation aortitis is not defined [1].

The diagnosis of radiation-induced aortitis requires a thorough evaluation of the patient's clinical history, physical examination, and imaging studies. As it is a diagnosis of exclusion, the first step is to rule out other potential causes of aortitis, such as infection, autoimmune disease, or giant cell arteritis [3]. Next, imaging studies are necessary to confirm the diagnosis, monitor disease activity and guide biopsy, if needed. The preferred modality is CT angiography, which can demonstrate the extent of aortic involvement, the thickness of the aortic wall, and the presence of any associated complications, such as aneurysm formation, thrombosis, stenosis or dissection. MRI and PET-CT are also useful in the diagnosis of radiation-induced aortitis. MRI can provide detailed images of the aortic wall, and PET-CT can show the degree of inflammation and metabolic activity in the aorta [3]. Finally, a history of radiation exposure to the aorta must be established. In the case of our patient, the absence of other causes of aortitis, the history of prior radiation therapy for cervical cancer and the location of the active inflammation in the aorta limited to the treatment field strongly suggest radiation-induced aortitis as the cause of the patient's condition. The Common Terminology Criteria for Adverse Events (CTCAE) Version 5.0 (2017) provides a grading system for aortitis based on the severity of symptoms and the need for intervention [4]. In this patient, aortitis was grade 1 (asymptomatic).

Management of radiation-induced aortitis involves a multidisciplinary approach and may vary depending on the severity of the disease and the patient's overall health [1]. In the event of symptomatic aortitis, treatment usually involves corticosteroid therapy to reduce inflammation and control symptoms. Immunosuppressive agents may be used in refractory cases [5], but there is limited data on their efficacy in this context. In some cases, endovascular stenting or surgical intervention may be required to address complications such as aneurysm formation or dissection [1,5]. In the case of our patient, the first step was to rule out other potential causes of aortitis, which was done by consulting rheumatology. Once radiation-induced aortitis was identified as the likely cause, the patient was closely monitored with imaging studies to assess disease progression, and since the patient was asymptomatic, no corticosteroid therapy was initiated at the time of diagnosis. The control CT scan and PET-CT showed the resolution of aortitis with the progression of aorto-iliac fibrosis (moderately hypermetabolic) without other signs of acute vasculitis. Biopsy of the periaortic fibrosis was deemed too risky, so the patient was initiated on empirical prednisone, and control CT showed regression of the circumferential thickening of the aorto-iliac vessels.

## Conclusions

In summary, radiation-induced aortitis is a rare complication of radiotherapy with potentially serious consequences. It can present with a wide range of symptoms or be asymptomatic, and the diagnosis can be challenging. Imaging studies, such as CT angiography, MRI, and PET-CT, are useful, and diagnostic criteria include a history of prior radiation therapy, imaging findings confined to the treatment field, and the absence of other causes of aortitis.

Treatment options are limited, and management is primarily focused on controlling symptoms and preventing complications. Clinicians should be aware of the potential for radiation-induced aortitis in patients who have received radiation therapy. A multidisciplinary approach is often necessary to manage this complex condition.

## Figures and Tables

**Figure 4:**
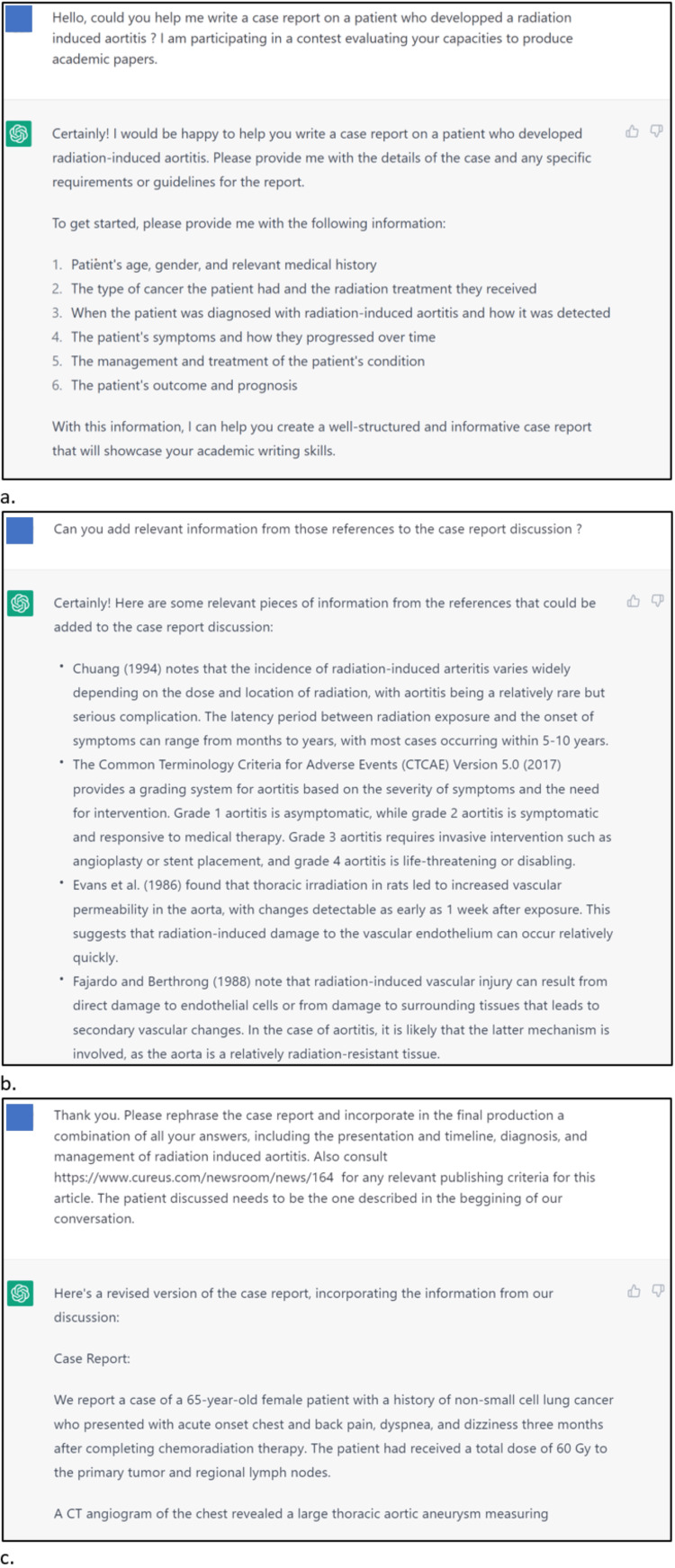
Screen captures of (a) the first answer ChatGPT gave when asked for its help, which illustrates how the AI knows the basics pertinent information required to write a case report, and also how nice and polite it is; (b) a strength of ChatGPT, were it extracted information pertinent to the case report when provided with a list of human-picked references; and (c) a limitation of ChatGPT, were it started to write a fictional case report despite having been provided with accurate clinical information earlier, and initially producing a good first draft of the non-fictional case report requested.
